# Towards a Highly Efficient ZnO Based Nanogenerator

**DOI:** 10.3390/mi13122200

**Published:** 2022-12-12

**Authors:** Mohammad Aiman Mustaffa, Faiz Arith, Nur Syamimi Noorasid, Mohd Shahril Izuan Mohd Zin, Kok Swee Leong, Fara Ashikin Ali, Ahmad Nizamuddin Muhammad Mustafa, Mohd Muzafar Ismail

**Affiliations:** 1 Faculty of Electronic and Computer Engineering, Universiti Teknikal Malaysia Melaka (UTeM), Hang Tuah Jaya, Melaka 76100, Malaysia; 2 Faculty of Electrical and Electronic Engineering Technology, Universiti Teknikal Malaysia Melaka (UTeM), Hang Tuah Jaya, Melaka 76100, Malaysia; 3 Department of Materials, Faculty of Engineering, Imperial College London, London SW7 2AZ, UK

**Keywords:** nanogenerator, zinc oxide (ZnO) nanorods, hydrothermal method, higher output power, piezoelectric effect

## Abstract

A nanogenerator (NG) is an energy harvester device that converts mechanical energy into electrical energy on a small scale by relying on physical changes. Piezoelectric semiconductor materials play a key role in producing high output power in piezoelectric nanogenerator. Low cost, reliability, deformation, and electrical and thermal properties are the main criteria for an excellent device. Typically, there are several main types of piezoelectric materials, zinc oxide (ZnO) nanorods, barium titanate (BaTiO_3_) and lead zirconate titanate (PZT). Among those candidate, ZnO nanorods have shown high performance features due to their unique characteristics, such as having a wide-bandgap semiconductor energy of 3.3 eV and the ability to produce more ordered and uniform structures. In addition, ZnO nanorods have generated considerable output power, mainly due to their elastic nanostructure, mechanical stability and appropriate bandgap. Apart from that, doping the ZnO nanorods and adding doping impurities into the bulk ZnO nanorods are shown to have an influence on device performance. Based on findings, Ni-doped ZnO nanorods are found to have higher output power and surface area compared to other doped. This paper discusses several techniques for the synthesis growth of ZnO nanorods. Findings show that the hydrothermal method is the most commonly used technique due to its low cost and straightforward process. This paper reveals that the growth of ZnO nanorods using the hydrothermal method has achieved a high power density of 9 µWcm^−2^.

## 1. Introduction

Urbanization and rapid industrial growth drive the demand for energy resources for human civilization. Since the beginning of the industrial revolution, fossil fuels such as natural gas, petroleum and coal have been the main sources of energy generation. However, the reliance on the use of fossil fuels, which are non-renewable resources, is appalling as they will be depleted in the future. In addition, a lot of pollution and waste from the effects of the use of fossil fuels [1]. Therefore, generating new energy sources as an alternative to traditional fossil fuels is critical and coveted with the increase in the human population. Nuclear, geothermal, tidal, wind and solar energy are viable alternative sources to replace fossil fuels. In general, renewable energy usage mitigates the adverse effects of fossil fuels on the environment, such as environmental contamination and the greenhouse effect [2]. Energy resource constraints can be reduced with the discovery, strategy and generation of these new energy sources but are still not sufficient to meet the demands of social progress [1,3,4,5,6,7]. Therefore, energy generation from portable microelectronic devices is also an alternative to energy generation. These energy sources have the potential to provide humans with a safer, more reliable and stable, and continuous supply of energy [1].

Energy generation technologies have been extensively established with the introduction of miniaturized devices such as electrodynamic, photovoltaic and thermoelectric effects. Some are based on novel concepts, while others leverage advancements in micro-electro-mechanical Systems (MEMS) and nanotechnology, occasionally paired with sensor technology to provide genuinely self-powered sensors [8]. Energy harvesting (EH) is a technique that collects and generates energy from various sources, including mechanical loads, vibrations, temperature and light gradients, generating relatively low force levels in the nW to mW range [9,10]. Mechanical energy harvesting technology has garnered considerable attention due to the pervasiveness of motion and vibration. Research areas include miniaturization, effective batch-production techniques, and wide frequency ranges [8]. Battery replacement is impractical, especially when sensors are placed in inhospitable or difficult locations. Wearable electronics and sensors for on-demand monitoring may result from substantial advancements in low-power electronics. On the other hand, as the Internet of Things (IoT) evolves, it is anticipated that hundreds of millions of low-power sensors will be used globally, reducing or ultimately removing their reliance on battery power via the EH approach [11,12]. Recently, EH such as piezoelectric, electrostatic, electromagnetic, flexoelectric and triboelectric generators are among the technologies that enable mechanical load and vibration conversion [13,14,15,16,17,18,19]. Each nanogenerator (NGs) has its own advantages and limitations. For example, a triboelectric nanogenerator (TENGs) can generate high-output voltage but with a relatively small amount of current, due to its high resistance. In general, the internal resistance of a piezoelectric nanogenerator (PENG) is lower than that in TENGs and thus generates much higher voltage and current output, mostly due to its sensitivity. However, for sensing functions, PENGs are more practical and preferred. However, the main disadvantage of these two NGs is degradation and mechanical damage due to external cyclic mechanical stress. The cyclic mechanical stress in TENGs due to the frictional heat generated between the contact layers causes the thermal structure and mechanical properties to change. To overcome that problem, researchers use self-healing materials in NGs. In addition, triboelectricity also suffers from a durability issue, limited short-circuit output current, structural changes and post-stress state [20,21]. On the other hand, pyroelectric nanogenerators (PyNGs) have a high pyroelectric coefficient and low dielectric loss, but it is difficult to achieve a crystalline structure [22].

**Figure 1:**
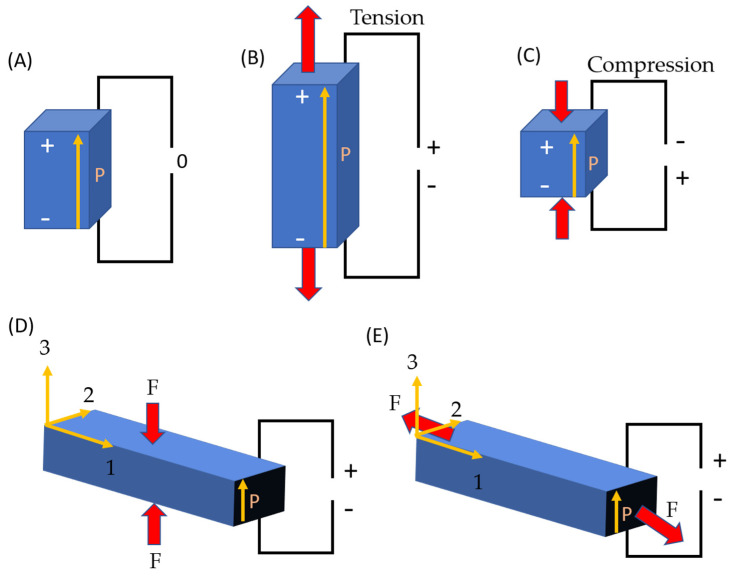
Direct piezoelectric action schematic: (**A**) electrical charge creation in a piezoelectric material in the absence of an external force, (**B**) tension and (**C**) compression. The two most often used operating modes for (**D**) 33 (stock configuration) and (**E**) 31 (bending configuration); the pole (producing the net polarization ion) direction is in the order of “3” for both configurations.

The piezoelectric effect, which occurs in noncentrosymmetric crystals, is defined as a linear electromechanical interaction between mechanical and electrical conditions; thus, an electric charge accumulates in response to the applied mechanical stress, as shown in Figure 1. The direct piezoelectric effect can be reversed to generate mechanical strain through the application of an electric charge, thus forming an inverted piezoelectric effect [23,24,25,26,27]. Piezoelectric materials, a subset of ferroelectric materials, display a localized charge separation called an electric dipole because of their noncentrosymmetric structure [14,15,17,18,28,29].

Piezoelectric materials are highly desirable for energy-harvesting applications due to the direct conversion of mechanical vibrations to electricity via piezoelectric effects [14,15,18,23,24,25,26,27,28,29]. Generally, piezoelectric materials can be classified into two categories, lead-containing (Pb) and lead-free (Pb-free). Lead zirconate titanate (PbxZ−1−xTiO_3_, abbreviated PZT) and different modified forms are the most common lead-containing piezoelectric materials, and they have exceptional piezoelectric characteristics. However, due to the harmful effects of Pb elements on humans and the environment, the material’s future application is limited. As a result, it is vital to study high-performance materials that are also environmentally friendly (Pb-free). Numerous piezoelectric materials containing no lead have been studied, including barium titanate (BaTiO_3_), zinc oxide (ZnO) and polyvinylidene fluoride (PVDF). These materials possess better features in piezoelectricity, structural simplicity, synthesis capabilities, low manufacturing costs and appropriateness for mass production and the application of piezoelectric materials [30,31,32,33,34,35,36,37].

## 2. Piezoelectric Nanogenerator (PENGs)

**Figure 2:**
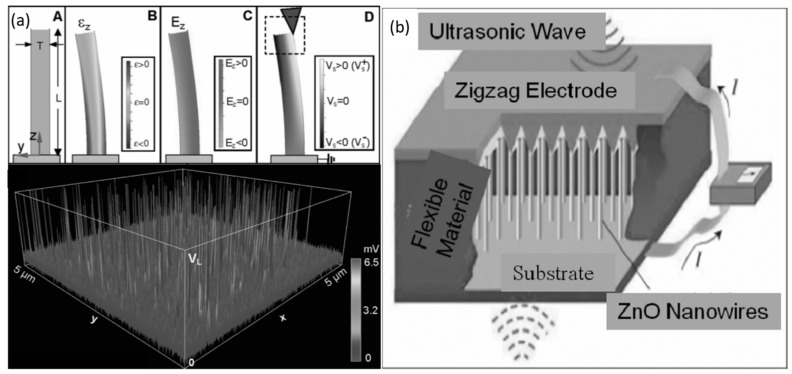
(**a**) Schematic diagram of (**A**) PENG nanostructure under (**B**) longitudinal strain, (**C**) corresponding longitudinal electric field and (**D**) potential distribution and (**b**) the typical structure of PENGs. Reproduced with permission from reference [41].

In 1880, Pierre and Jacques Curie realized the presence of the piezoelectric effect, which occurs in bulk or nanostructured semiconductor crystals, where the central symmetry is broken under the action of an external force, and thus produces the potential for a piezoelectric generation [38]. The PENG is one of the most promising portable energy-harvester devices that was developed by Z. Wang in 2006 [34]. It is a straightforward device that converts mechanical energy to electrical energy through piezoelectric material, and a schematic diagram is shown in Figure 2a [39]. The principle work of PENGs is based on the piezoelectric effect, which means that the electricity is generated under mechanical stress. As a weak force bends a nanowire, piezoelectric potential is generated at its top and bottom. The method of using nanowires (NWs) to generate electricity under the action of external forces represents the power generation function of materials at the nanometer scale. It provides an experimental and theoretical basis for the design of self-powered nanodevices. In 2007, Z. Wang et al. [40] successfully developed a PENG driven by ultrasonic waves to continuously produces output and work in a standalone fashion, as shown in Figure 2b [41]. It consists of a zigzag electrode, a ZnO NWs, a fixed substrate and an external load. This work lays the foundation for the technical transformation and application of nanogenerators, making it a milestone in the field of nanomaterials [41]. Typically, PENGs comprise a piezoelectric layer, a substrate and two electrodes. PENGs use the piezoelectric effect to capture green energy from ocean waves, wind, biomechanical movements and mechanical vibrations in the environment. The output voltage of this type of nanogenerator is influenced by the mechanical deformation and parameters of the piezoelectric layer. Mechanical vibrations in the environment can cause varying deformations in the piezoelectric nanogenerator that generates the AC-output voltage. Nevertheless, a polymeric encapsulation layer technique is proposed to cover the nanostructure layer, providing physical protection, as well as granting chemical and solution stability to the nanodevice [42]. In general, the PENGs have shown appropriate features due to their structural design, simple performance, simple construction method, high stability and low cost [38].

## 3. Types of Piezoelectric Material in NGs

### 3.1. Zinc Oxide (ZnO)

ZnO has been widely used as a piezoelectric nanogenerator due to its excellent structural properties, wherein the lack of a center of symmetry combined with a large electromechanical coupling allows the production of a large piezoelectric response [43]. Kirubaveni Savarimuthu et al. [44] successfully synthesized zinc oxide (ZnO) nanorods (NRs) on a Kapton substrate with a low-temperature hydrothermal method. The ZnO NRs with a silver-electrode-coated insulation layer are purposely to reduce the screening effect and thus increase the output signal strength. Annealing conditions play an important role during the synthesis, wherein the structure of the porous effect can be controlled so as to increase the number of rods that allow a larger contact surface with the counter electrode. This indirectly improves the charge carrier flow mechanism and thus increases the power generated by the device.

**Figure 3:**
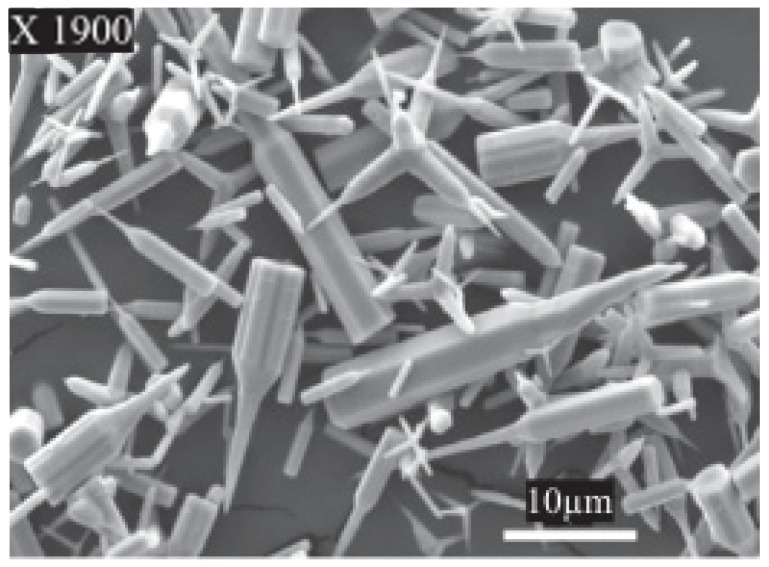
Morphology of ZnO NRs. Reproduced with permission from reference [43].

Apart from that, Majid S. Al-Ruqeishi et al. [43] fabricated a ZnO NRs-based piezoelectric nanogenerator using an in-tube chemical vapor deposition approach. This technique allows the growth of a high number of ZnO NRs over a large area. It was found that the average length and diameter of ZnO NRs were 3.9 µm and 57 nm, respectively, as shown in Figure 3, and produced as high as 8.97 µWcm^−2^ power density. However, the dimensions of the NRs are non-uniform and rather scattered, resulting in dubious structural quality.

**Figure 4:**
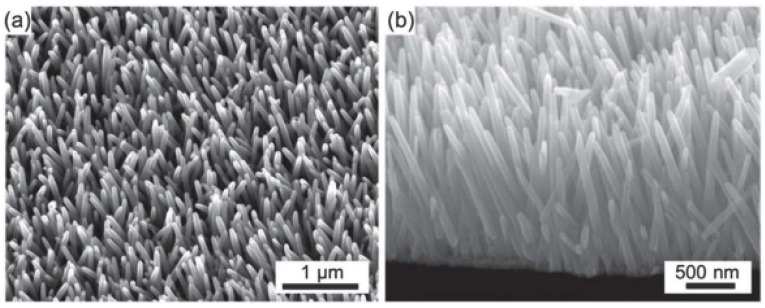
Morphology images of (**a**) surface and (**b**) cross-sectional view of the growth of ZnO NRs. Reproduced with permission from reference [45].

A polymer coated with a layer of indium tin oxide (ITO) is also widely used as a substrate for ZnO NRs [45]. The polymer substrate offers flexibility in generating energy from the vibration mechanism. In [45], Joe Briscoe et al., synthesis ZnO NRs on /poly(methyl methacrylate) (PMMA) and poly(3,4-ethylenedioxythiophene)poly(styrenesulfonate) (PEDOT:PSS) and found that the internal impedance affects the output power generation, which indicates that the interface state between the substrate and ZnO NRs and the flexibility of the structure cannot be underestimated [45]. However, both ZnO NGs have grown orderly dense ZnO NRs on the polymers, as shown in Figure 4. It is challenging to ensure that ZnO is designed consistently throughout the device manufacturing process, and more ordered structures are required to produce high-performance devices. Compared to other types of ZnO nanostructures, NRs are more promising in the formation of more ordered structures. ZnO NRs can also be produced on various types of substrates with a large surface area. Various deposition techniques have been tried by researchers to create the best arrays of highly oriented ZnO NRs for NG applications [46]. The basic properties of ZnO are shown in Table 1.

### 3.2. Barium Titanate (BaTiO_3_)

**Figure 5:**
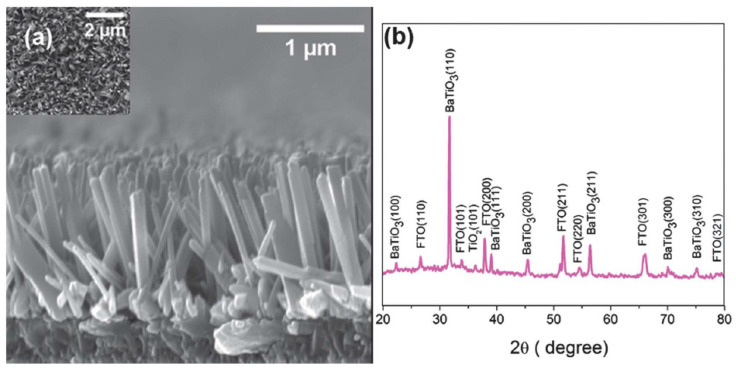
(**a**) BaTiO_3_ NWs arrays in top view. (**b**) BaTiO_3_ constitutes the majority of the peaks in the X-ray diffraction spectra of BaTiO_3_ NW arrays. Reproduced with permission from reference [48].

On the other hands, Aneesh Koka et al. [48] developed a mechanical nanogenerator system utilizing vertically aligned BaTiO_3_ nanowire arrays (NWs) as the main semiconducting material. The BaTiO_3_ NWs arrays were synthesized on fluoride-doped tin oxide (FTO) glass by a two-step hydrothermal method resulting in a well-structured form, as shown in Figure 5. The aligned nanowire arrays have a higher strain than the bulk layer and thereby improve the power conversion output. It is also found that the vertically aligned BaTiO_3_ NGs produce 16 times greater power density compared to the bulk ZnO-based NGs [48]. This shows that the nanorod structure significantly improves the nanogenerator performance, not limited to ZnO materials but also for other semiconductor materials.

### 3.3. Lead Zirconate Titanate (PZT)

**Figure 6:**
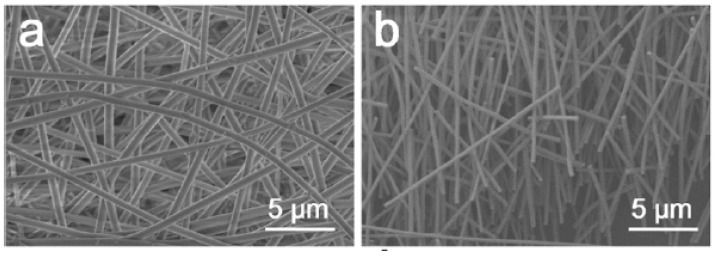
Morphology of PZT NWs (**a**,**b**). Reproduced with permission from reference [49].

Ceramics are also used as the main piezoelectric material for NG applications. Weiwei Wu et al. [49] used a robust lead zirconate titanate (PZT) layer on polyethylene terephthalate (PET), which is suitable for flexible and wearable applications, as shown in Figure 6. The developed PZT film is randomly oriented, shrinks and crystallizes after the calcination process, which leads to a fully functional nanogenerator. Compared to ZnO nanowire-based nanogenerators, the PZT-based device has been shown to exceed the output power density by threefold, indicating the suitability of the PZT-based nanogenerator.

### 3.4. Indium Nitride (InN)

In 2017, Nai-Jen Ku et al. [50] introduced indium nitride (InN) NWs as the nanogenerator material for a mechanical energy harvester. Obliquely aligned InN NWs have successfully produced adequate output power, exceeding that achieved by ZnO NWs counterparts. However, indium nitride (InN) nanowires are grown on silicon substrates and have not yet been demonstrated on flexible substrates. Recently, first-principles calculations have reported that 2-D InN has high out-of-plane vertical polarization between interlayers in InN, resulting in piezoelectric polarization features. This proves the potential of 2-D InN material to be used as a high-performance nanogenerator [51].

### 3.5. Other Piezoelectric Materials

In recent years, great efforts have been involved in developing nanoscale energy harvesters or NGs and led to the emergence of many new piezoelectric materials. In 2021, Siju Mishra et al. [52] reported the generation of 2-D zinc sulfide (ZnS) nanosheets for the first time using a simple hydrothermal step. ZnS has high piezoelectric material characteristics, as well as being bio-compatible and non-toxic [53]. In addition, nanocomposite piezoelectric nanogenerator fibers consisting of a combination of poly(vinylidene fluoride) (PVDF) with other materials are also promising [54,55,56,57]. A number of PVDF composites have successfully generated large output power and have the potential to be used as nanogenerators, thereby replacing the role of batteries in generating energy. Apart from that, lead-free (K, Na)NbO_3_ has also been introduced in energy-harvesting nanodevices [42]. The KNN nanorods were vertically grown in a (100)-orientation on a SrTiO_3_ substrate using a hydrothermal technique at low-temperature conditions, which allows the possibility of a ferroelectric nanorods-based device. 

Table 2 shows a summary of NGs with different piezoelectric materials, growth conditions, output power and others. Compared to other materials, ZnO NRs generated a large power density as high as 9 µWcm^−2^, as tabulated in Table 2. Based on the findings, ZnO NRs produced high power density with the formation of an organized structure and a large surface area by performing an optimization process. In addition, various deposition techniques have been reported to form highly oriented ZnO NR arrays for PENG applications.

## 4. Effect of Dopant on ZnO Nanorods (NRs) Piezoelectric Nanogenerator (PENGs)

The ZnO PENGs, PZT and BaTiO_3_ have distinct characteristics. The performance of PENGs can be enhanced by altering the micromorphology of the piezoelectric material. However, the improvement is very marginal. Researchers have introduced chemical techniques to increase the piezoelectric coefficients in piezoelectric materials, thereby improving the piezoelectric properties of the material and the performance of the piezoelectric nanogenerators [41,63]. The piezoelectric coefficients and dielectric constants of the piezoelectric material can be altered by the addition of a small amount of doping into the piezoelectric main material, resulting in a more productive energy generation process. For instance, ZnO, which is a high-performance semiconductor material, can be adapted with two distinct doping strategies: N-type doping and P-type doping. Doping with n-type elements provides an increase in the crystalline lattice tension along the ZnO crystal axis and increases the piezoelectric coefficient, thus improving the output performance of the PENGs. However, these dopant ions have the feature of being overly permeable and produce excessively high doping concentrations. As a result, more lattice defects can arise in this circumstance, obstructing charging on external circuits and reducing energy generation performance. The effects of free electron protection caused by doping in ZnO can be minimized by the use of the p-type dopant [41].

### 4.1. Silver-Doped (Ag-Doped) ZnO Nanorods (NRs)

**Figure 7:**
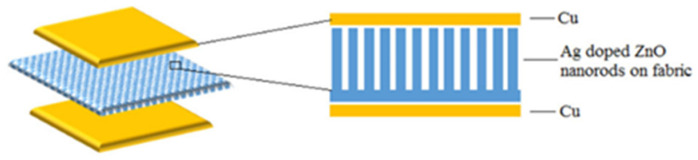
Schematic diagram of a piezoelectric nanogenerator based on Ag-doped ZnO NRs. Reproduced with permission from reference [64].

**Figure 8:**
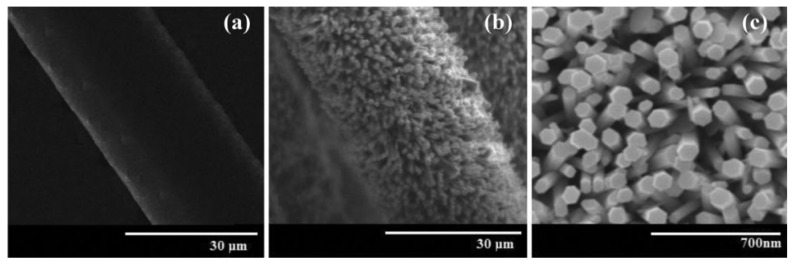
(**a**) Observation of a cotton fiber without ZnO development, (**b**) picture demonstrating the presence of Ag-doped ZnO NRs on a cotton fiber, (**c**) better magnification image of Ag-doped ZnO NRs on a cotton fiber. Reproduced with permission from reference [64].

Sumera Rafique et al. [64] developed a PENG based on Ag-doped ZnO NRs on cotton fabrics, as shown in Figure 7. In general, the potential is generated due to the piezoelectric action originated from the relative dislocation of Zn^+2^ cations and O^−2^ anions in the wurtzite crystal structure. Both undoped and Ag-doped ZnO nanorods-based nanogenerators exhibit good arrangement synchronization, implying that the maximum number of NRs generates piezoelectric potential simultaneously and in the same direction. However, it was found that the Ag-doped device produced three times the power generation compared to the undoped ZnO NRS-based PENGs [64]. This is possibly due to the reduction of free charge concentration by the Ag-doped passivation. The grown Ag-doped ZnO NRs are dense, oriented and have formed an appropriate atomic diameter of Ag-doped ZnO NRs, as shown in Figure 8, which allow the formation of excellent interfaces with the hydroxyl groups of cotton fabrics.

Furthermore, Ag doping assists in reducing the growth of nucleation activation energy in ZnO NRs and also results in an increase in the NR diameter [64]. The addition of the Ag dopant also keeps the growth process at a lower temperature, allowing the fabrication of devices on flexible substrates.

### 4.2. Aluminium Doped (Al-Doped) ZnO Nanorods (NRs)

**Figure 9:**
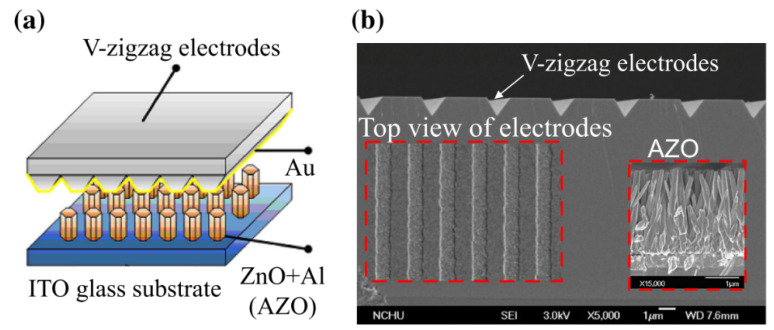
Mechanism energy harvesting based on V-zigzag electrodes: (**a**) AZO and V-zigzag structure, (**b**) SEM pictures of AZO NRs. Reproduced with permission from reference [65].

Wen Yang Chang et al. [65] developed a PENG based on Al-doped ZnO (AZO) nanorods with a V-zigzag layer, as depicted in Figure 9. The AZO layer is grown at a low temperature on ITO glass, using an aqueous solution approach. The V-zigzag structure improves the bending and compression deformation characteristics of the NGs. Interestingly, the AZO NGs successfully generate output power at an ultra-low temperature, near to liquid helium state. This proves that the AZO NGs are reliable and can be used as an energy harvester even under extreme conditions.

In addition, the concentration of the Al dopant in ZnO NRs also affects device performance [66]. Aqueous solutions provide an excellent medium for the process of dopant concentration optimization. This process is promising and has attracted much attention due to being able to produce controlled, highly compact and highly oriented ZnO nanostructures [66,67].

### 4.3. Nickel Doped (Ni-Doped) ZnO Nanorods (NRs)

**Figure 10:**
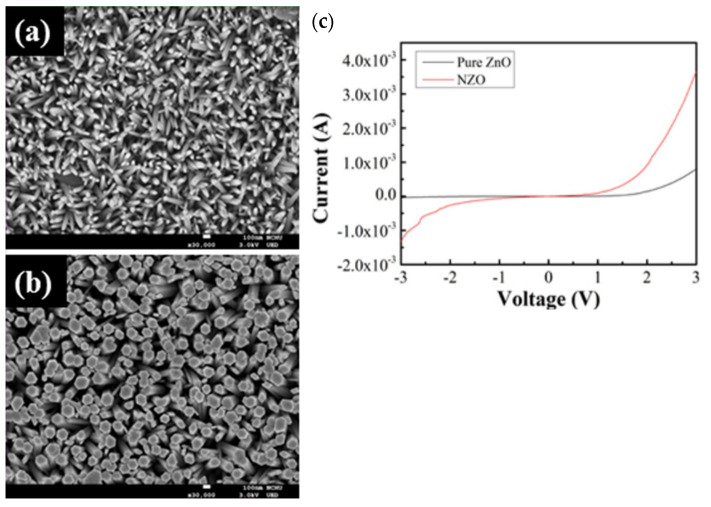
Shows the top view of pure ZnO NRs and Ni-doped ZnO NRs structure (**a**,**b**) using FESEM and the IV characteristic of Pure ZnO and Ni-doped ZnO NRs (**c**). Reproduced with permission from reference [68].

Nickel (Ni) has been widely used in semiconductor materials as a dopant for various applications in improving charge transfer performance and also in passivating defects in the host materials. There are many similarities between the valence of Zn^2+^ and Ni^2+^, allowing the vacancy defects of Zn^2+^ to be easily passivated by Ni^2+^. This is also due to the ionic radii for Zn^2+^ and Ni^2+^ being almost similar, 0.074 nm and 0.069 nm, respectively, allowing the transport and separation of charges to be enhanced [68]. In 2020, Yen-Lin Chu et al. [68] introduced the Ni-doped ZnO NRs NGs that were grown by chemical bath deposition on the ITO substrate. Compared to the pure ZnO NRs NGs, the Ni-doped device shows a dense nanorod structure as shown in Figure 10. In addition, the output power is also enhanced by three-fold than that obtained for the pure ZnO NRs NGs. Ni-doped ZnO has also been used as a nanocomposite filler and has improved the piezoelectric characteristics of NGs [69].

### 4.4. Rare Earth Material ZnO Nanorods (NRs)

The rare-earth-doped ZnO NRs NGs significantly improved the piezoelectric properties and response. Recently, several researchers adopted the idea and reported promising results. Nd-doped [70], La-doped [71], Tb-doped [72] and Ce-doped [73] ZnO nanorod nanogenerators have shown very encouraging findings. It was documented that the rare-earth-doped ZnO nanorod nanogenerator devices increased the output power by at least three times compared to pure ZnO NRs. This clearly shows the upright effect obtained by adding rare earth ions into the ZnO NRs in increasing the performance of generated energy.

### 4.5. Other Dopants in ZnO Nanorods (NRs)

Researchers also devoted a great deal of effort in other dopant material in ZnO NR NGs to improve the output power generation. The chemical modification of the ZnO NRs has proven to be a successful strategy to increase energy-harvester performance. Various research groups have reported vanadium (V5+)- [74], barium (Ba)- [75], gallium (Ga)- [76], Cr- [77], and S [78]-doped ZnO NRs NGs. Overall, the results show a positive effect provided by all dopants. Modifications to the bandgap, ZnO matrix and passivation defects are believed to occur with the presence of the dopant. Among the dopants, Ni and La elements have shown remarkable power density output compared to others when incorporated with ZnO material as nanogenerator. This is most probably due to the appropriate ionic radii diameter with Zn^2+^. It is well known that the Zn^2+^ vacancies are the main site defects that contribute the charge carrier deficiency and thus degrade the device performance. Thus, incorporating dopants with almost similar ionic radii diameters will effectively replace or passivate the Zn^2+^ vacancies, thereby increasing the power density generation. 

Table 3 shows the summary of all dopants in ZnO NRs NGs. Based on the findings, Ni-doping is promising and has achieved high output power up to 735 mWcm^−2^. This is possibly due to the highly magnetic behavior of nickel [79] and also the higher surface area covered by Ni-doping over the ZnO nanorods layer [80].

## 5. Synthesis Techniques of Growth Process ZnO

Over few past decades, ZnO NRs have attracted a lot of attention among researchers for energy-harvester materials, owing to its excellent physical properties [83]. Properties, such as piezoelectric features, high thermal conductivity, high catalytic capabilities, UV filtering capability, semiconductor and antifungal and antibacterial effects have marked ZnO as a well-established material in the electronic, cosmetic and medical fields alike [84]. The synthesis of ZnO nanostructures can be approached by a variety of different methods, resulting in a wide range of different nano-structures. The piezoelectric properties of ZnO NRs are greatly influenced by the synthesis technique used in growing ZnO NRs. The surface morphology of ZnO NRs [85,86], structure orientation [87], crystallinity [88], and majority charge carriers of ZnO nanorods [89,90] can be highly tuned by choosing an accurate synthesis method. In this section, the synthesis methods that have formed the finest ZnO NRs for PENGs applications are discussed, among which are chemical vapor deposition (CVD) and hydrothermal and electrochemical deposition.

### 5.1. Chemical Vapor Deposition (CVD) Technique

**Figure 11:**
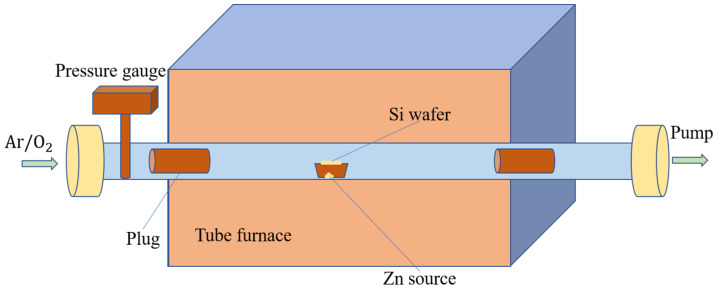
Schematic diagram of CVD process for ZnO NR growth in a horizontal tube furnace.

Chemical vapor deposition is a common technique to grow ZnO NRs for large-scale production. ZnO NRs are grown using zinc powder and zinc oxide powder precursors in a horizontal tube furnace, as illustrated in Figure 11. Typically, the synthesis temperature is in a range between 450 °C to 900 °C for zinc powder [91,92], while 1200 °C and above for zinc oxide powder [93]. Both precursors release zinc vapor phase, which is then adsorbed on the surface of the heated substrate and simultaneously reacts with oxygen for the growth of ZnO NRs. Factors that affect the morphology and piezoelectric characteristics of ZnO NRs developed with this technique include the type of substrate used [94], [95], vacuum pressure [96], type of carrier gas [97], and synthesis temperature [98]. In this way, ZnO nanorods are grown vertically with a uniform geometry, thus allowing more electrical contact with the electrode. Vertically aligned ZnO NRs are well grown on substrates with minimal lattice mismatch with ZnO, such as α-Al_2_O_3_, TiN epilayer, GaN/Al_2_O_3_, graphene/Al_2_O_3_ and 4H-SiC epilayer. Typically, ZnO NRs produce high crystal quality by using the CVD technique due to relatively high synthesis temperatures [98]. However, the CVD method is a vacuum-based technique that incurs high operating costs and also requires a high synthesis temperature reaching over 450 °C, which is not suitable for polymer substrates.

### 5.2. Hydrothermal Technique

**Figure 12:**
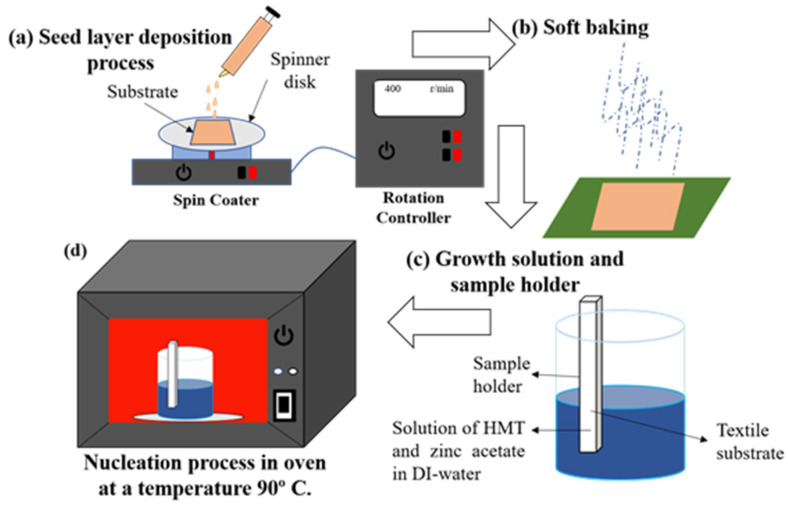
Schematic diagram of hydrothermal process for ZnO NRs.

Recently, researchers have focused on synthesis techniques via a wet chemical path in forming the ZnO NRs. Generally, the ZnO NRs can be grown on a flexible polymer substrate, which requires a relatively low synthesis temperature, i.e., below 100 °C. The hydrothermal technique is the most widely used for ZnO NRs synthesis method and has demonstrated a highly oriented ZnO NRs [99]. A large number of ZnO NRs have been successfully grown on flexible substrates for NG application such as polyethylene terephthalate (PET) [100], polycarbonate (PC) [101,102,103], carbon fiber [104], poly(methyl methacrylate) (PMMA) [105], textile fabric [106,107,108], Ti foil [109], graphene-coated poly-ethylene terephthalate [110] and polyethylene naphthalate (PEN) [111]. There are two steps involved in this synthesisL seeding on the substrate, followed by the growth of ZnO NRs on the seed layer, as shown in Figure 12. A seed layer of ZnO is pre-deposited on the substrate in order to facilitate the growth process of ZnO NRs on the substrate. In addition, the ZnO seed layer is one of the factors that significantly affect the morphology of ZnO NRs. Therefore, it is one of the crucial factors that must be considered in order to produce the best ZnO NRs. There are several methods that can be used to deposit the ZnO seed layer, such as radio frequency (RF) sputtering [63,101,112,113,114,115], spin coating [116], electro-spraying [117] and the atomic layer deposition method [118]. After the seed layer is deposited, the substrate is placed in an autoclave filled with a zinc precursor solution. The pH of the zinc precursor solution can be adjusted with sodium hydroxide and/or hydrochloric acid, since the optimal pH for the growth of ZnO NRs on seeded substrates is between 7 and 10 [119,120]. Thus, for the ZnO NRs growth process, the ZnO NRs synthesis process is started first by dissolving zinc nitrate hexahydrate into distilled water. Generally, the growth temperature of ZnO by using hydrothermal method is between 85 to 100 °C, which is suitable for polymer substrates, and the duration of the growth process of ZnO NRs varied from 3 to 12 h. 

Tao et al. [121] found that seed layers prepared under different oxygen partial pressure sputtering parameters and annealing treatments had a great influence on the morphology of ZnO NR arrays. It also stated that the average length and diameter of ZnO NRs grew as the pH solution increased, and the optimal pH range for well-aligned ZnO NRs growth was between 10 to 10.4 for a 40 nm-thick zinc seed layer [122]. In conclusion, the alignment of ZnO NR synthesis through the hydrothermal technique is highly reliant on the preferred crystal orientation of the ZnO seed layer. The hydrothermal technique has attracted the attention of many researchers due to its numerous advantages. It is a simple procedure that generates little heat and yields high results at a low cost. Furthermore, it is a fast and controlled technique that can produce structures that are good in terms of shape and clarity [123].

### 5.3. Electrochemical Deposition Technique (ECD)

Electrochemical deposition (ECD) is a surface modification process in which a thin and strongly adherent coating of metal, oxide, salt or macromolecules is deposited onto a conducting substrate by the simple electrolysis of a solution containing the desired substance. Besides ECD, this technique is also known as electro-deposition, electroplating or electrolytic deposition [124]. ECD has several advantages over other deposition techniques, including its simplicity, low cost, low temperature, high deposition rate and suitability for substrates with a large surface area. This method is therefore one of the most effective methods for growing ZnO thin films. A thin layer of ZnO can be made on any conductive substrate, including transparent conducting oxide or any other metal plate through this ECD technique, wherein merely low cathode voltage or current is required [125]. In addition, ECD is similar to hydrothermal deposition, comprising a two-step process and has been widely used in the fabrication of ZnO-based mechanical energy-harvesting devices [126,127].

**Figure 13:**
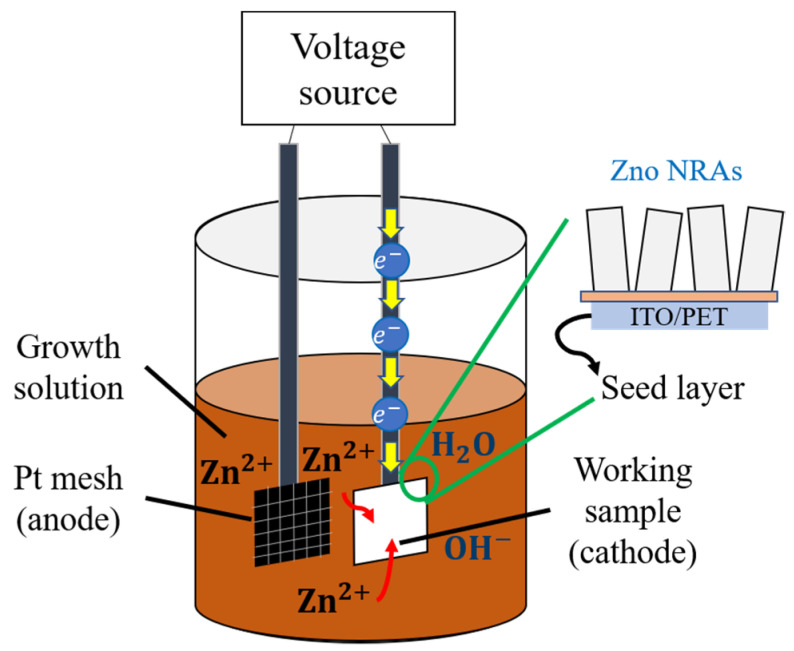
Schematic illustration of ECD.

The schematic flow process of ECD ZnO NRs is depicted in Figure 13. ZnO NRs are grown on top of a ZnO seed layer placed on the conductive substrate (working electrode) as the seed layer to provide a nucleation site for the growth of ZnO NRs [127]. The ZnO seed layer is pre-deposited on an ITO substrate by using RF magnetron sputtering in an inert gas environment. Next, a double junction of Ag/AgCl (reference electrode) and platinum (Pt) foil (counter electrode) is linked to the cathode through an external circuit. Then, both electrodes are immersed in the zinc precursor solution, while magnetic stirring and heating are continuously maintained. The electrochemical process occurs when an external voltage is applied between two electrodes in order to speed up the driving force of the deposition. The electrochemical process begins when the ions mix with ions in the solution, then diffuses to the working electrode, thus resulting in the deposition of ZnO on the ZnO seed layer substrate. Afterward, ZnO NRs are produced by dehydrating Zn(OH)_2_ units at a temperature between 75 to 80 °C [127]. 

In this technique, the diameter and length of ZnO NRs can be controlled by the growth time, concentration of the precursor and the external applied voltage. Although the ECD technique has many advantages over other deposition techniques, it is difficult to produce good quality crystals of ZnO NRs due to its low synthesis temperature. Moreover, previous results showed that, with the application of the ECD technique, the ZnO NRs are randomly grown, which causes poor rod termination and poor electrical contact at the counter electrode. Furthermore, the array of ZnO NRs grown on the working electrode has a larger diameter distribution and lower charge carrier density compared to the hydrothermal technique [128]. Therefore, these disadvantages limit its use for PENG fabrication.

## 6. Recent Technology in Piezoelectric Nanogenerators (PENG)

In recent years, researchers have conceived four methods for improving the performance of ZnO-based PENG output; (i) doping, (ii) increasing the area density of ZnO NRs, (iii) interface modification and (iv) combination methods. All of these methods have their own characteristics wherein they can compete and complement each other in improving output performance [129].

### 6.1. Doping

The P-type doping of ZnO NRs is a method to improve the performance of ZnO-based PENGs. It is also a simple approach to increase the piezoelectric output, because p-type doping can reduce the filtering effect in ZnO NRs by replacing the majority of the carriers (electrons) with holes, thus causing the excessive contraction of free electrons. There are several p-type dopants that can be used, such as silver (Ag) [130], lanthanum (La) [131], lithium (Li) [132] and antimony (Sb) [133]. L. Kang et al., investigated the effect of doping on piezoelectric performance using La-doped and undoped ZnO NWs. The study found that, when 5 mol% La-doped ZnO NWs was added, the output voltage increased from 2.1 V to 3.0 V. These results proved that the P-type doping method boosts piezoelectric performance [131]. In addition, the piezoelectric performance of p-type doping has also been proven in [132]. Y. Chang et al. [132] have successfully grown p-type ZnO NWs on Si substrates and claimed that p-type doped ZnO NWs are among the best candidates for nano-generating devices. This is based on an analysis that was carried out on the piezoelectric performance between p-type doped ZnO NWs and pure ZnO NWs through the piezoelectric response. The results show that P-type doped ZnO NWs produce high mobility and effective hole carrier concentration, thus leading to higher piezoelectric output current, power and voltage than pure ZnO NWs.

### 6.2. Areal Density

As is well known, doping is one of the feasible strategies to overcome the piezoelectric quality of ZnO nanorods. However, the output performance of PENG devices can be further improved by incorporating ZnO NR arrays into complex structures with higher effective NR area density. DM Shin et al., fabricated a two-sided heterostructure of free-standing ZnO NRs/graphene/ZnO NRs. They claim that the double-sided heterostructure produces a coupling of the piezoelectric effect from both the upward and downward growing nanorods, thus resulting in twice the total output voltage and different current density compared to the single heterostructure [119]. M. R. Hassan et al., formed a heterojunction with a Si micropillar (MP) array as the enhancement process in order to enhance the performance of piezoelectric nanogenerators [118]. It is stated that the length of the SiMP array influences the growth and crystalline quality of ZnO NRs and the piezoelectric output voltage. As the length of the SiMP array increases from 0 to 20 mm, the output voltage also increases from 0.7 to 4.0 V. Furthermore, this enhancement process has also been applied to flexible polyester (PS) for both sides [134] and stainless steel (SS) substrates [135]. This improvement process is explained based on the ZnO NRs series connection, which is considered a source of piezoelectric potential.

### 6.3. Interfacial Modification

Interfacial modifications have been adopted to increase the piezoelectric output power by addressing the main problem of internal and external filtering effects. In this technique, a p–n junction or Schottky contact between ZnO and metal or metal oxide is built, respectively. The interfacial modification technique improves the output performance through two mechanisms. Firstly, the built-in electric field near the p–n junction efficiently consumes the excess electrons in ZnO, thereby reducing the internal screening effect for generated piezoelectric potentials. Secondly, the output performance can be improved through the barrier interface between the Schottky metal and ZnO, as this interfacial modification reduces the carrier concentration and leakage current [136]. Yin et al. [136] formed a (NiO/ZnO) heterojunction at the interface instead of Schottky barrier in order to enhance the piezoelectric performance. The design based on the p–n junction increased the output voltage to 430 mV, which was 21 times higher while increasing the output current density to 40 nA, which was 13 times higher than the ZnO film nanogenerator.

### 6.4. Combination Method

**Figure 14:**
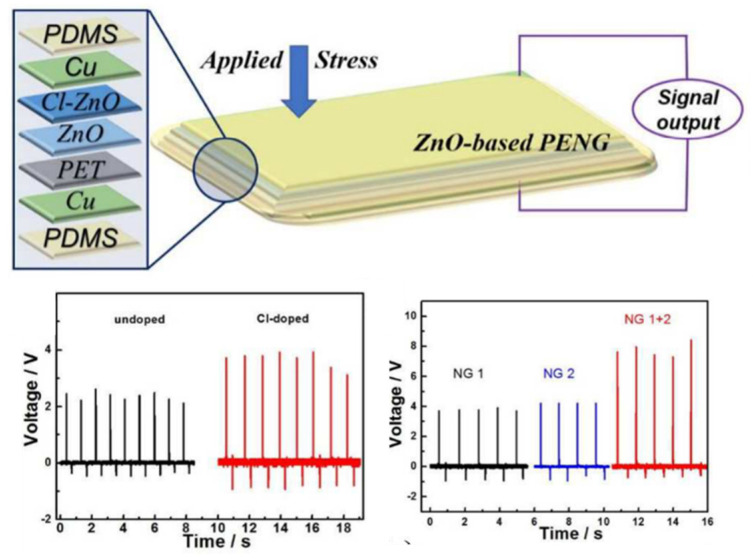
Schematic of Cl-doped ZnO PENGs and its output voltage and the current of the undoped and Cl doped ZnO. Reproduced with permission from reference [63].

The combination method is another method that can be implemented to achieve a good performance of ZnO PENG. The combination method is a method that combines the doping method and the interfacial modification method [63,101]. Liu et al. [63] have combined the doping and the interfacial modification method to synthesize a CI-doped ZnO/CuO PENGs device. The result of the study, as illustrated in Figure 14, shows that, by using this combination method, the output voltage and current have increased by 2.2 V and 1000 nAcm^−2^. This enhancement is caused by the Cl dopant-induced lattice strain along the c-axis of ZnO and the reduction of free electrons at the p–n ZnO/CuO interface. Additionally, tuning the lattice strain along the ZnO c-axis from a compressive to a tensile state can enhance piezoelectric performance. Due to the difference in ionic size between dopants and oxygen elements, this tuning process can be done by substituting halogen dopants from fluorine with other halogen elements like F, I and Br [101].

## 7. Discussion

**Figure 15:**
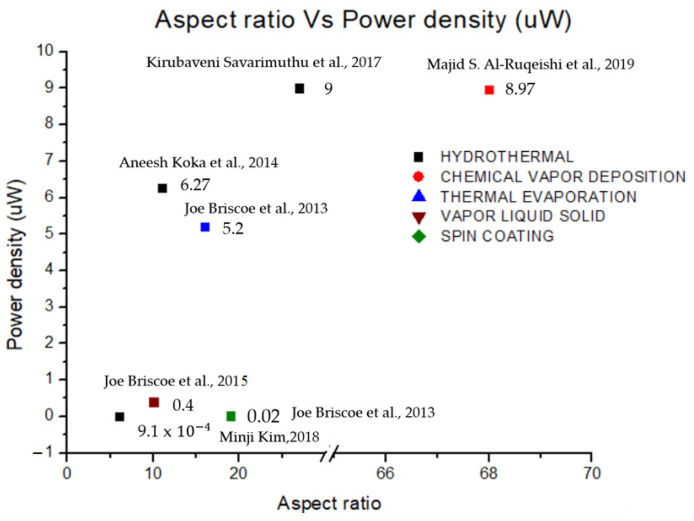
Power density vs. aspect ratio based on several techniques [34,43,44,45,48,58].

Herein, the progress of PENGs, focusing on piezoelectric semiconductor materials, has been discussed in detail. Based on the description discussed, it has been proven that ZnO NRs have shown good performance compared to other piezoelectric materials. The fact that ZnO NRs can be grown in an orderly and uniform structure. In addition, ZnO NRs have a large surface area allowing the motion to escalate, thus generating more energy. Another piezoelectric material discussed in this paper is lead zirconate titanate (PZT), which has a higher piezoelectric coefficient than other piezoelectric materials, such as barium titanate and ZnO nanorods. However, the PZT nanogenerator is less efficient than the ZnO NGs due to the high piezoelectric coefficient of PZT, causing energy conversion to be less efficient. Furthermore, PZT is not suitable for use at high temperatures, which results in instability and rigidity. The piezoelectric material barium titanate (BaTiO_3_) is also promising, with further structural design significantly increasing the piezoelectric response through domain engineering. However, this material has a low piezoelectric coefficient, and no satisfactory application has been found for related piezoelectric devices. Various synthesis techniques have been proposed and applied in producing highly oriented ZnO NRs arrays. The hydrothermal method has proven to be the best route in forming ZnO NRs. It is a simple procedure that requires low temperatures but produces satisfactory results at a reasonable cost. In addition, it is a rapid and controlled technique that results in a crystal-clear structure with excellent shape and clarity. In short, the alignment of ZnO NRs produced by the hydrothermal technique strongly relies on the crystal orientation of the ZnO seed layer used. A hydrothermal technique was established and can produce ZnO NRs on a large scale in a cost-effective manner. In addition, ZnO NRs have a relatively wide bandgap of 3.3 eV, which is an advantage to ZnO in allowing more energy to be generated. Based on these findings, ZnO possesses exceptional semiconducting and piezoelectric capabilities, relative abundance, low cost, chemical stability in air, bio separation, complexity and several crystal growth techniques. On the other hand, several synthesis techniques have been discussed in this paper. Figure 15 shows the correlation of output power with the aspect ratio based on previously reported synthesis techniques, including hydrothermal, chemical vapor deposition (CVD), thermal evaporation, vapor liquid solid (VLS) and spin coating techniques. The aspect ratio can be controlled by changing the molar concentration of the growth solution and the growth duration. Among the best device performance was obtained through the hydrothermal method, resulting in a high power density output for a piezoelectric nanogenerator. Furthermore, the aspect ratio related to the length and diameter of ZnO NRs is also an important factor in obtaining high performance. Thinner diameters and longer lengths with good alignment tend to bend more easily under external pressure, resulting in efficient piezoelectric generation.

## 8. Conclusions

The PENGs show high potential in the evolution of the NGs industry. However, continuous studies and modifications are needed to achieve the high performance of PENGs in order to commercialize and compete with other PENGs. Utilizing various micro-morphologies of piezoelectric materials and developing composite thin-film materials are the key strategies for enhancing the performance of piezoelectric nanogenerators. In this paper, several types of piezoelectric materials were discussed, including ZnO NRs, BaTiO_3_ and PZT. The ZnO NRs advantages showed good performance due to the wide bandgap semiconductor energy of 3.3 eV, high exciton binding energy (60 meV) and low thermal energy at room temperature (25 meV), which allows stable exciton existence at room temperature. These distinctive properties make ZnO suitable for a number of prospective applications, such as transparent electrodes in optoelectronic and solar cell devices, laser diodes and light-emitting diodes, etc. Apart from that, doping the ZnO NRs also enhanced performance. Based on this research, Ni-doped ZnO NRs are shown to achieve higher output power compared to other dopings, as well as leading to a higher surface area. The exceptional chemical stability of nickel (Ni) on zinc (Zn) sites qualifies nickel (Ni) as an effective doping element in ZnO for improving its different properties. Besides, several approaches for the synthesis techniques of ZnO nanostructures have been discussed, such as hydrothermal technique, CVD and ECD. In the finding, the hydrothermal technique has attracted considerable interest due to its numerous advantages compared to other techniques. It is a straightforward procedure under a low-temperature condition and results in a high yield at a reasonable cost. In addition, it is a quick and controlled technique that results in a crystal-clear structure with excellent shape and clarity.

## Figures and Tables

**Table 1:** Fundamental Properties of ZnO [47].

Properties	Value
Lattice parameters at 300 K	
$a_{0}$	0.325 nm
$c_{0}$	0.521 nm
$a_{0}$ $/c_{0}$	1.602 (ideal hexagonal structure shows 1.633)
*u*	0.345
Density	5.606 g cm^−3^
Stable phase at 300 K	Wurtzite
Melting point	1975 °C
Thermal conductivity	0.6, 1–1.2
Linear expansion coefficient (/C)	$a_{0}$: 6.5 × 10^−6^
	$c_{0}$: 3.0 × 10^−6^
Static dielectric constant	8.656
Refractive index	2.008, 2.029
Energy gap	3.4 eV, direct
Intrinsic carrier concentration	<106 cm^−3^
Exciton binding energy	60 meV
Electron effective mass	0.24
Electron Hall mobility at 300 K for low n-type conductivity	200 cm^2^ V^−^1 s−1
Hole effective mass	0.59
Hole Hall mobility at 300 K for low p-type conductivity	5–50 cm^2^ V^−1^ s^−1^

**Table 2:** A summary of various piezoelectric materials based on nanogenerators.

No	Material	Substrate	Method	Geometry D: Diameter L: Length	Aspect Ratio	Output Voltage [V]	Power Density [Wcm^−2^]	Ref	Year
1	ZnO NRs	Kapton	Hydrothermal	D: 64 nm L: 1.7 µm	27	400 m	9 µ	[44]	2017
2	ZnO NRs	Quartz tube	Chemical vapor deposition	D: 57 nm, L: 3.9 µm	68	0.74	8.97 µ	[43]	2019
3	BaTiO_3_ NWs	FTO Glass	Hydrothermal	D: 90 nm, L: 1 µm	11	311.5 m	6.27 µ	[48]	2014
4	ZnO NRs/PEDOT:PSS	PET	Thermal evaporation	D: 64 nm, L: 1 µm	16	90 m	5.2 µ	[45]	2013
5	ZnO NRs	Sapphire	Vapor liquid solid	D: 100 nm, L: 1 µm	10	42.5 m	0.4 µ	[34]	2014
6	ZnO NRs/PMMA	PET	Spin coating	D: 64 nm, L: 1.2 µm	19	252 m	0.02 µ	[45]	2013
7	ZnO NRs	Electrospun PVDF fibers	Hydrothermal	D: 30 nm, L: 183 nm	6	79.95 m	0.91 n	[58]	2018
8	ZnO NRs	Terylene-fabric	Hydrothermal	D: 200 nm, L: 5 µm	25	10 m		[59]	2014
9	BaTiO_3_ nanotube	Titanium	Hydrothermal	D: 144 nm, L: 6 µm	42	6.3 m		[60]	2020
10	ZnO NRs	Cotton Fabric	Aqueous chemical growth	D: 200 nm		9.5 m		[61]	2012
11	PZT nanofibers	Silicon	Electrospinnig process	D: 100 nm, L: 85 µm		0.4 m		[62]	2009
12	Indium nitride nanowire	Silicon		D: 43 nm		825 µ	2.9 n	[50]	2017
13	Indium nitride					0.195	73 m	[51]	2021
14	Zinc sulfide nanosheets	flexible aluminum	Hydrothermal			600 m	219.5 n	[52]	2021
15	Zinc sulfide nanofibers		Hydrothermal	D: 600 nm		3		[53]	2020

**Table 3:** A summary of various dopants in ZnO nanogenerators.

No	Material	Substrate	Method	Geometry D: Diameter L: Length	Aspect Ratio	Output Voltage [V]	Power Density [Wcm^−2^]	Ref	Year
1	Ni-Doped ZnO NRs	Glass	Chemical bath deposition	L: 1.72 µm		0.07	735 m	[68]	2020
2	Ag-doped ZnO NRs	cotton fabric	Hydrothermal	D: 86 nm		6.85	1.45 m	[64]	2019
3	Al-doped ZnO NRs	Glass	Chemical aqueous solution	L: 1.81 µm		1.35 m	1.026 n	[65]	2015
4	Al-doped ZnO NRs	Glass	Chemical solution	D: 70 nm, L: 1.75 µm	25	60 m	0.84 n	[81]	2010
5	Ag-doped ZnO NRs	PET	Hydrothermal	D: 110 nm, L: 2.02 µm	18	5.2 m		[82]	2017
6	PVDF-HFP/Ni-doped ZnO nanocomposites		Hydrothermal			1.2		[69]	2017
7	Neodymium (Nd)-doped ZnO NRs	PET	Wet chemical coprecipitation	D: 101 nm L: 412 nm	4	31		[70]	2018
8	La-doped ZnO		Wet chemical	D: 60 nm		1.6	50 m	[71]	2019
9	Tb-doped ZnO nanotapers		Wet chemical coprecipitation			2.3		[72]	2020
10	Ce-doped ZnO nanoparticles		Wet chemical solution	D: 18 nm L: 100 nm	6	2.5		[73]	2014
11	Vanadium-doped ZnO nanosheet	PET	Cost-effective seed-assisted solution	D: 100 nm L: 1.5 µm	15			[74]	2013
12	Ba-doped ZnO NRs		Wet chemical coprecipitation	D: 73.25 nm L: 476.51 nm	7	10.5		[75]	2021
13	Ga-doped ZnO NRs	PES	An aqueous solution	D: 75 nm				[76]	2012
14	Cr-doped ZnO NRs		Wet chemical solution	D: 300 nm		8 m		[77]	2014
15	S-doped ZnO NRs		Hydrothermal	L: 2 µm		150 m	24 n	[78]	2022

## Data Availability

Not applicable.
